# Persistent Lymphadenopathy due to IgG4-Related Disease

**DOI:** 10.1155/2012/158208

**Published:** 2012-11-12

**Authors:** Benjamin Smith, Matthew B. Carroll

**Affiliations:** 81st Medical Group Hospital, 301 Fisher Street, Keesler Air Force Base, MS 39534, USA

## Abstract

A 28-year-old healthy female presented to her primary care physician with lymphadenopathy, fatigue, malaise, and night sweats. Symptoms persisted despite conservative treatment and eventually the patient underwent multiple lymph node resections and a bone marrow biopsy before a diagnosis of IgG4-related disease (IgG4-RD) was made. IgG4-RD is a relatively new disorder first histopathologically recognized within the last decade. As the disease can affect a single organ or multiple organs, symptoms can vary greatly among patients. With symptoms ranging from mild, such as lower extremity edema, to severe, such as spinal cord compression, IgG4-RD must be considered in appropriate patients. Diagnostic criteria have been proposed based on organ involvement, serum IgG4 levels, and histopathological criteria. Diagnosis can be difficult to make with many studies suggesting different values for diagnostic criteria, such as the level of tissue IgG4+/IgG+ cell ratio to delineate IgG4-RD. Treatment consists of high dose glucocorticoids as a first line therapy with some patients choosing instead to simply undergo observation. This case illustrates the difficulty in diagnosis and the need for increased awareness among medical professionals.

## 1. Introduction

IgG4-RD is a relatively newly classified condition with unifying histopathologic features recognized only since the early 2000s [1]. The disorder is thought to mainly affect middle-aged to elderly men with a median age of onset of 58 years old [2, 3]. Exact incidence and prevalence are difficult to determine as many cases most likely go undiagnosed with limited awareness currently amongst medical professionals. Clinical findings vary by the organ system involved but many times are associated with tissue swelling or enlargement [1]. Due to the variety of clinical presentations, diagnosis can be delayed months to years and result in unnecessary tests and procedures.

## 2. Case Presentation

**Figure 1:**
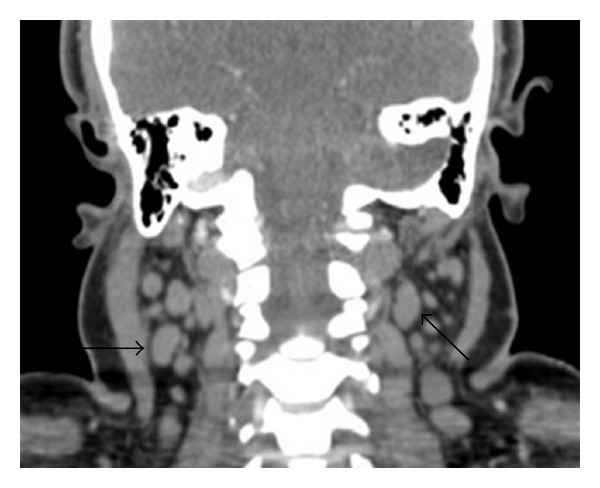
CT neck with contrast revealed diffuse cervical adenopathy (arrows demonstrate lymphadenopathy).

**Figure 2:**
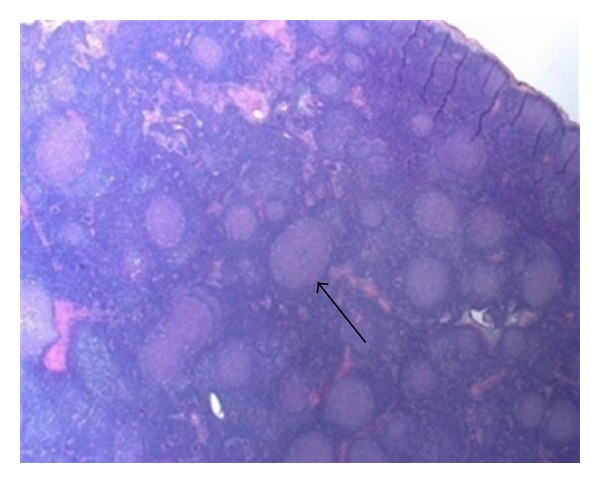
Hematoxylin and eosin stain of a resected lymph node, 2x magnification. This preparation shows nonspecific reactive follicular hyperplasia (arrow demonstrates secondary follicle with prominent germinal center).

A 28-year-old female with no significant past medical history presented to her primary care physician with lymphadenopathy, night sweats, fatigue, and malaise. She was treated empirically and partially responded to a short course of glucocorticoids and an antibiotic. When initial laboratory workup was unrevealing for an obvious etiology and symptoms persisted, computed tomography (CT) of the neck and chest with contrast was performed that showed diffuse adenopathy (Figure 1) and hepatosplenomegaly. The patient was referred for lymph node resection that initially revealed only nonspecific B cell proliferation and did not support a specific etiology (Figure 2). With lymphoma a concern, she was referred to Hematology/Oncology for assessment. Hematology/Oncology evaluated the patient and performed a bone marrow biopsy which showed no cellular abnormalities consistent with lymphoma or leukemia. The patient's symptoms then stabilized for several months. 

**Figure 3:**
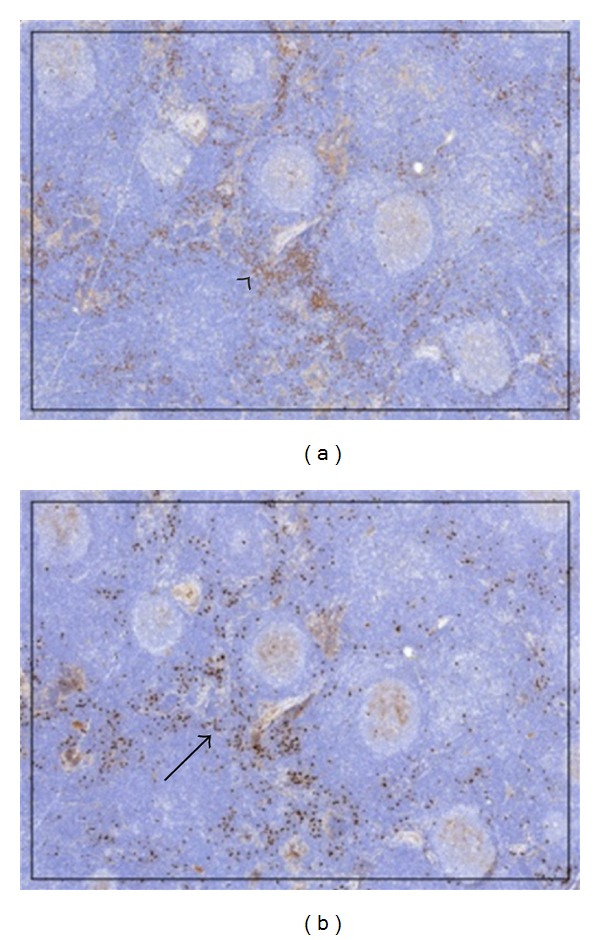
IgG (a) and IgG4 (b) stains, 4x magnification. These stains show an IgG4+/IgG+ cell ratio estimated at 30% (black arrow head: IgG staining, solid black arrow = IgG4 staining).

However, when her symptoms again worsened, she was referred to Infectious Disease who entertained a broad differential including Kikuchi's syndrome, Epstein-Barr virus (EBV), cytomegalovirus (CMV), toxoplasmosis, or human immunodeficiency virus (HIV) infection. Extensive serological testing was unrevealing and a second lymph node was removed, again without an obvious etiology. Rheumatology was then consulted and expanded the differential to include systemic lupus erythematosus, Sjögren's syndrome, Castleman's disease, autoimmune lymphoproliferative syndrome, and IgG4-related disease (IgG4-RD).  Table 1 highlights the various serologic and tissue examinations performed as a part of the workup for this patient. An additional lymph node was removed and sent to Pathology for IgG4 staining (Figure 3) which, in combination with the patient's elevated serum IgG4 level of 206 mg/dL (normal 4–86 mg/dL), was very suspicious for IgG4-RD. Treatment modalities offered to the patient included a prolonged course of glucocorticoids or rituximab but she declined therapy as she and her spouse were attempting to conceive. The patient continues to attempt conception and therefore treatment has still not been initiated.

## 3. Diagnosis

The diagnosis of IgG4-RD can be difficult to make and misdiagnosis is common. One set of proposed diagnostic criteria as outlined in Table 2 calls for (1) organ enlargement, mass or nodular lesions, or organ dysfunction; (2) a serum IgG4 concentration >135 mg/dL; (3) histopathological findings of >10 IgG4 cells/high powered field (HPF) with an IgG4+/IgG+ cell ratio >40%. Based on these criteria a diagnosis is “definite” in patients who fulfill criteria (1), (2), and (3), “possible” if criteria (1) and (2) are met, and “probable” in patients with criteria (1) and (3) satisfied. The situations in which these criteria prove most difficult to utilize are in those from whom tissue cannot safely be obtained or it is very difficult to biopsy the involved organ, such as in pancreatic disease. When the diagnosis is in question, further clarification can be sought using organ specific criteria for IgG4-RD, such as in autoimmune pancreatitis, Mikulicz's disease, and IgG4-related kidney disease. These additional organ-specific criteria greatly increase the specificity of the diagnostic criteria in applicable settings [2]. 

While the diagnostic criteria discussed earlier suggest an IgG4+/IgG+ cell ratio of >40%, other proposed diagnostic criteria recommend more stringent requirements such as a ratio of >50%. Others suggest that some cases of IgG4-RD may present with an even lower ratio such as >30% [1, 4]. A retrospective study performed by Masaki et al. looked at the various cutoffs and how this would alter the sensitivity and specificity of the diagnostic criteria, with results shown in Table 3. 

Another aspect of the proposed diagnostic criteria state the serum IgG4 concentration should be >135 mg/dL. However, the serum IgG4 level can be misleading, with approximately 30% of patients with otherwise classic findings for this disease having normal serum IgG4 concentrations [1]. Conversely, the serum IgG4 level can be elevated in a variety of other unrelated conditions including primary sclerosing cholangitis, many forms of nonautoimmune pancreatitis, and atopic dermatitis. Even in the normal population, an elevated IgG4 level can be found in up to 5% of those tested [5].

Tissue biopsy and evaluation play a central role in diagnosis. Although IgG4-RD can manifest in nearly any organ system, it shares unique histologic findings regardless of location including dense lymphoplasmacytic infiltrate rich in IgG4-positive plasma cells, storiform fibrosis which is that of an irregularly whorled pattern, obliterative phlebitis, and an eosinophilic infiltrate [1, 6]. While the histology is characteristic, diagnosis requires specific immunohistochemical confirmation with IgG4 immunostaining. A finding of >10 IgG4 cells/HPF has benefits and shortcomings. In one study, setting the cutoff at >10 IgG4 cell/HPF provided 100% sensitivity but only 38.1% specificity [4]. Poor specificity has also been documented in various other studies that have revealed high numbers of IgG4 cells/HPF in other disease states, such as in cases of granulomatosis with polyangiitis [7]. 

Diagnosis can be difficult but the most important first step is a high clinical suspicion. This disease requires specific testing to be recognized and therefore must be sought out, not revealing itself on routine labs or biopsy. Furthermore, because of some shortcomings in the current diagnostic criteria, one must thoroughly consider other disorders in the differential diagnosis. In our case of unexplained generalized lymphadenopathy, fatigue, and night sweats, the differential diagnosis included various infections (EBV, CMV, toxoplasmosis, HIV, and mycobacterium), lymphoma/leukemia, metastatic neoplasia, systemic lupus erythematous, Castleman's disease, autoimmune lymphoproliferative syndrome, and Sjögren's syndrome [8]. However, with a thorough evaluation to exclude alternative diagnoses, in the setting of our patient's elevated serum IgG level and significant staining for IgG4, her diagnosis was established.

## 4. Treatment

Treatment strategies have not yet been validated by large randomized controlled trials. Recommendations are currently limited to case reports and consensus statements, with glucocorticoids considered first. Several glucocorticoid sparing regimens have also been suggested, with rituximab showing efficacy even in refractory disease [1, 6]. Treatment should include the input of the patient, as sometimes a watch-and-wait approach is sufficient depending on symptoms and organ involvement [1]. Regardless, patients should be monitored closely, because the disease process can progress and extensive fibrosis can occur making a treatment response less likely. Our patient chose a watch-and-wait approach as medication side effects were unacceptable to her and her husband as they were trying to conceive a child. 

## 5. Conclusion

IgG4-RD is a new disease that has been present for many years but only better appreciated within the last decade. The currently proposed diagnostic criteria incorporate organ involvement, the serum IgG4 level, and tissue evaluation with IgG4+/IgG+ ratio and IgG4 cells/HPF. These criteria must be interpreted in the right clinical setting and diagnosis made only after a thorough consideration and exclusion of alternative diagnoses. Clinical features of the disease can be difficult to piece together given the wide variety of potential systems that can be involved and, therefore, the physician must have a high clinical suspicion. Treatment based on case reports and consensus consists mainly of glucocorticoids or similar agents that suppress lymphocytes. As the disease definition and our understanding of it continues to evolve, greater physician awareness is needed so that this diagnosis is not overlooked in future patients. Ongoing study of patient's with IgG4-RD will provide a better understanding of this disorder, how to best approach treatment, and potentially a more refined diagnostic scheme.

## Figures and Tables

**Table 1:** Differential diagnosis with pertinent laboratory findings*.

Lymphoma	Bone marrow and lymph node biopsy performed HTLV-1+2 antibodies (nonreactive)
Castleman's disease	HHV-8 lymph node stains negative
Autoimmune Lymphoproliferative syndrome	Less than 1.5% double negative (CD4−CD8−) T-cell count on cytometry
Mycobacterial infection	AFB culture of lymph node (negative)
Systemic lupus erythematosus	ANA panel (1 : 160)
	Anti-dsDNA and anti-Sm antibodies (negative)
	Complement levels C3, C4 within normal range
Viral infections	EBV (nuclear Ag IgG positive, viral capsid IgG positive, viral capsid IgM negative),
	CMV (IgM negative, IgG negative), parvovirus B19 (IgM negative, IgG negative),
	Hepatitis (HBs Ag negative, HBc Ab negative, HBs Ab negative, HCV Ab negative)
Cat scratch disease	Bartonella panel (negative)
Sjögren's syndrome	SSA/SSB (negative)

∗HTLV-1+2: human T-lymphotropic virus type I and II, HHV-8: human herpes virus 8; AFB: acid-fast bacilli, ANA: antinuclear antibodies, Anti-dsDNA: antidouble-stranded DNA, Anti-Sm: anti-Smith; EBV = Epstein-Barr virus, CMV: cytomegalovirus, HBs Ag = hepatitis B surface antigen, and HBc Ab: hepatitis B core antibody IgG, HBs Ab = hepatitis B surface antibody, HCV Ab: hepatitis C virus antibody, SSA: anti-Ro/SSA antibodies, and SSB: anti-La/SSB antibodies.

**Table 2:** Proposed comprehensive diagnostic criteria for IgG4-RD*.

(1) Organ enlargement, mass or nodular lesions, or organ dysfunction	
(2) A serum IgG4 concentration >135 mg/dL	
(3) Histopathological findings of >10 IgG4 cells/high powered field and an IgG4+/IgG+ cell ratio >40%	
Definite = (1), Possible = (1) + (2), Probable = (1) + (3)	

*Adapted from Umehara et al. [2].

**Table 3:** Specificity and sensitivity of IgG4+/IgG+ cell ratio for diagnosis of IgG4-RD*.

IgG4+/IgG+ Cell ratio	Sensitivity	Specificity
>30%	100%	71.4%
>40%	94.4%	85.7%
>50%	94.4%	95.2%

*Adapted from Masaki et al. [4].
